# Quality of Life of Post-renal Transplant Patients in Rawalpindi

**DOI:** 10.7759/cureus.33083

**Published:** 2022-12-29

**Authors:** Naafiah K Mallick, Amna Hassan, Rai Salaar Sultan Bhatti, Daneyal Rafique, Ailiya R Jaffery, Imtenan Sharif, Noor U Zameer, Hamayun Mustafa

**Affiliations:** 1 Community Medicine, National University of Medical Sciences, Rawalpindi, PAK; 2 Internal Medicine, Combined Military Hospital (CMH) Lahore Medical College and Institute of Dentistry, Lahore, PAK; 3 Community Medicine, Army Medical College, National University of Medical Sciences, Rawalpindi, PAK; 4 Internal Medicine, Army Medical College, National University of Medical Sciences, Rawalpindi, PAK

**Keywords:** public health, who-qol bref, pakistan, renal transplant, quality of life

## Abstract

Objectives

The objective is to assess the overall quality of life (QoL) in patients who had undergone renal transplant within the last three years and correlate this index with various demographic variables such as age, gender, marital status, and education level and to correlate the QoL score calculated vs. the health status perceived by the patients themselves

Materials and methods

This was an analytical cross-sectional study, carried out over a period of five months. A total of 123 patients were targeted among which data from 79 patients were gathered including all the patients that underwent kidney transplantation in the past three years at a renal transplant center in CMH, Rawalpindi, Pakistan. Non- Probability convenience sampling was used, and data were collected using the WHOQOL-BREF tool that contained 25 questions targeted to four domains (physical, psychological, social, and environmental). The Questionnaire was administered over the phone with proper consent taken beforehand. Data were analyzed using Excel and SPSS version 23.

Results

A total of 79 patients were administered the questionnaire with the mean age of our study population being 35±11 years out of which 84.5% were male and 15.5% were female. Patients received the kidney from relative donors (98.4%) with the highest percentage being sister donors (30.9%). The majority of patients reported from Punjab (54.4%), with the rest from far-flung rural areas. An estimated 62.5% of the patients presented with other systemic/psychological disorders such as DM+, IHD, HTN, Hepatitis C, depression, etc. The mean global score of these patients was 79.21 which can be broken down into four domains, physical domain 80.40, psychological domain 78.99, social domain 82.70, and environmental domain 74.75.

Conclusion

In a developing country such as Pakistan, with most of the patients belonging to lower or middle socioeconomic groups, we believe that the patient’s own sense of QoL is overshadowed by the mere exuberance of being given a second chance at life which was portrayed by the discrepancies in the perceived vs actual QoL graph. One common recurring theme that was noticed whilst interviewing the patients was that the difficulties they might have faced post-transplant paled in comparison to how grateful they were to live another day. A positive trend was noticed between the time since transplant and the QoL score which could be attributed to various factors such as the use of aggressive immunosuppressants, fear of injury, fear of transplant rejection, etc. in the first-year post-transplant. Demographic variables such as income, age, location, etc. did not affect the scores of these patients on a great scale. The present study aims to guide clinicians in the improvement of long-term outcomes of renal transplantation in Pakistan.

## Introduction

World Health Organization defines the quality of life (QoL) as “an individual's perception of their position in life in the relation to cultural values of the system they live in along with their goal expectations, standards and concerns” [1]. QoL refers to the person’s wellbeing such that it covers four dimensions, which are physical, psychological, social, and environmental. Health status, functional status, and QoL are three interchangeable concepts that refer to the same field of “health”. The health field involves negatively valued aspects of life, including death, as well as more positively valued aspects such as complete satisfaction with one's health and wellbeing. The restrictions of definition usually depend on the reason that one is evaluating health and the specific concerns of patients, clinicians, and researchers [2]. QoL constitutes a personal and individual question that delivers itself to a theoretical rather than a scientific approach. It would, theoretically, be necessary to extend these scales to include psychological factors such as anxiety, depression, the ability to socialize, and the extent to which an individual achieves his/her expectations [3]. End-stage renal disease (ESRD) is defined as a permanent decline of kidney function in a person, which is severe enough to be fatal in the absence of dialysis or transplantation. ESRD is included under stage five of the National Kidney Foundation Kidney Disease [4]. ESRD affects more than 1500 people per million population in countries with a high prevalence (such as the US and Japan). About two-thirds of people with ESRD receive hemodialysis, one-quarter have kidney transplants, and one-tenth receive peritoneal dialysis. Risk factors for ESRD include progressive age; hypertension; diabetes mellitus; obesity and a history of renal disease [5]. Patients with ESRD may be treated with dialysis or renal transplantation (RTX). RTX is considered superior to chronic dialysis, as it prolongs survival and alleviates uremic symptoms [6].

In patients with ESRD, QoL is affected by the disease itself and by the treatment option that the patient resorts to. Numerous factors have been identified to affect the QoL in patients undergoing hemodialysis, for example, age [7], gender, duration of dialysis, family care and support, living status, patient compliance, education level [8], anti-depressants, hospitalization and gender and are associated with a lower QoL [9]. Elderly recipients had a significantly lower graft and patient survival as well as a significantly higher risk of graft loss and patient death [10]. Sleep and sleep disorders were not very common in renal transplant patients but higher than in the general population due to depression and lower education [11]. Life after renal transplantation presents with negative aspects as well, such as a strict regimen of immunosuppressive drugs and their related side effects, frequent medical visits, infections, the uncertainty and anxiety concerning rejection episodes, potential loss of the graft, lifetime cost, burden of the treatment and unemployment following the transplant.

In a developing country, such as Pakistan, patients may face communication gaps with their doctors due to a high population to medical personnel ratio [12]. Lack of transportation facilities also poses as a hurdle toward an optimum QoL, since high transportation costs and lengthy travel time may hinder the recovery of patients and may distort their normal day-to-day living conditions. Therefore, it is important to consider the personal, environmental, and clinical factors that negatively influence Health-related Quality of Life (HRQoL) outcomes in both categories. QoL can be helpful in determining the impact of interventions on the health of patients with chronic diseases when a cure is not possible. Various tools can be used to measure a patient's QOL such as WHOQOL-BREF, KTQ, SF-36, etc.

The purpose of this study is to assess the AoL of patients who have undergone renal transplants within the last three years. Through this study, we hope to evaluate not only the different domains that the patient is affected by but also the effect that the frame of mind of the patient has on the outcome of the transplant. We hope to evaluate the various perceptions held by Pakistani patients with regard to their health after transplant and the limitations they might face as opposed to the ones they actually face. By knowing the AoL of these patients and the common difficulties that they face in their day-to-day life, medical practitioners should consider these various factors in order to administer a healthcare regime more suited to each individual patient.

Renal transplants eliminate many limitations of dialysis and can improve the patient’s QoL. However, after transplantation, the patient must lead a controlled and disciplined life. There must also be regular follow-ups, the use of immunosuppressive medicine throughout the rest of the patient’s life, the possibility of infection, an increased chance of tumor growth, and the possible need for hemodialysis due to acute and chronic rejection. Physical psychological and social changes that transplantation caused in the patient has led to the discussion about the QoL of these people. People are no longer interested in the length of life; they are interested in the QoL [13].

A similar study conducted in India reported that QOL is significantly better in individuals who have undergone kidney transplants as compared to those who chose dialysis as a treatment option [14]. Patients in developing countries face greater obstacles because of the lack of transplantation centers, quality, safety issues, etc. [15]. Since Pakistan is a developing country, we expect the results to be similar to that of India as they are affected by similar physical and environmental domains. Not just developing countries but developed countries are also reporting a rising incidence of ESRD [16], which should bring our attention to factors contributing to this, and introduce the screening of the general population, especially diabetics and hypertensives so that they can resort to better treatment options, and to improve the QOL survival rates so that they can continue to live a normal, healthy life.

The purpose of replacement therapy is not only to prolong life expectancy or maintain good health but also to improve QoL. Assessment of QoL can open up effective and useful ways to provide care and treatment in the field of diagnosis, prognosis, and evaluation to help chronic disease patients and even healthy people. In this study, we aim to analyze the various factors that may have an impact on a patient's post-transplant QoL and to compare the patient's perceived vs actual QoL.

## Materials and methods

Study design

This was an observational cross-sectional study.

Setting

Patients from a renal transplant center in Rawalpindi were selected.

Duration of study

The study duration was five months from November 2018 to March 2019.

Sampling technique

It was a non-probability convenience sampling.

Sample size

A total of 123 patients were targeted who had undergone a renal transplant in the last three years amongst which data from 79 patients were gathered

Inclusion criteria

All the patients that underwent kidney transplantation in the past three years at a renal transplant center in Rawalpindi, Pakistan.

Exclusion criteria

Patients that denied consent, patients that had since settled abroad, patients that did not respond, and patients that had passed away.

Data collection procedure

The study was started after approval from the institutional ethics committee of Army Medical College. Data was collected using the WHOQOL-BREF tool that contained 25 questions targeted to four domains (physical, psychological, social, and environmental). The Questionnaire was administered over the phone with proper verbal consent taken beforehand. The questionnaire is graded from 0-100 for each domain, 0 being very poor QOL and 100 being a very good QOL score.

Data analysis

The Data was analyzed using Excel and SPSS version 25. The percentage and frequencies of qualitative variables were documented along with the mean and standard deviations of quantitative variables. A t-test was applied to compare means between the two groups and a p-value less than 0.05 was considered statistically significant.

## Results

**Figure 1:**
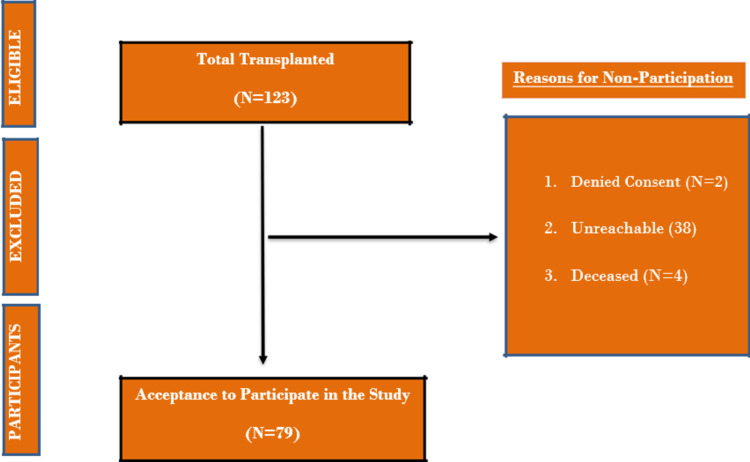
Sample selection

The characteristics of the sample are reflected in Figure 1. A total of 79 patients were administered the questionnaire, the mean age was 35±11 out of which 84.5% were male and 15.5% were females. 98.4% of the patients received the kidney from a relative donor with the highest percentage being sister donors (30.9%), 54.4% presenting from the Punjab region, with the rest from far-flung rural areas. 72.6% were married, with the remaining single, separated, etc. 50.6% of the sample had an educational status of above matriculate/O levels, with 9.8% being completely illiterate.

Around 62.5% of the patients presented with other systemic/psychological disorders such as DM+, IHD, HTN, hepatitis C, depression, etc. (7.60% reported with DM+, 14.00% with HTN). The median income of our study population was 39k Pak-rupees per month. An astounding 38.3% of the sample presented with a total family income of less than 30K Pak-rupees per month and were below the poverty line.

The average QoL scores based on questionnaire dimensions is shown in Table 1. It can be seen in the table that the lowest scores that the patients presented with were in the environmental domain which mainly amounted to questions 12, 13 and 24 that were aimed at the patient’s financial status, access to information, and transportation facilities, respectively.

**Table 1:** Domain scores

Questionnaire Dimensions	Quality of Life Score	Standard Deviation
Physical Domain	80.40	11.13
Psychological Domain	79.00	12.16
Social Domain	82.70	13.50
Environmental Domain	74.75	11.97
Global Score	79.21	9.33

Table 2 depicts the QoL scores with respect to age groups. There is no noticeable significant difference in age to the domain scores (p-value >0.05). The majority of the patients were <35 years of age and it is evident that the mean scores of this group are higher than those of the older age group. The widest gap between the two variables presents itself in the physical domain, where the older age group complained of overall decreased energy levels and difficulty in performing normal day-day activities due to lack of energy and fear of complications.

**Table 2:** Quality of life scores with age group. P-value tests the significance level between the subgroups (e.g., age- ≥35 and <35)

Domains	Age Group	Mean	Standard Deviation	P-value
Physical Domain	<35	82.86	11.64	0.100
	≥35	76.59	9.26	
Psychological Domain	≥35	78.82	12.67	0.877
	≥35	79.25	11.54	
Social Domain	<35	83.26	15.69	0.612
	≥35	81.83	9.34	
Environmental Domain	<35	76.15	12.12	0.194
	≥35	72.58	11.59	
Global Mean	<35	80.27	10.41	0.176
	≥35	77.56	7.22	

**Figure 2:**
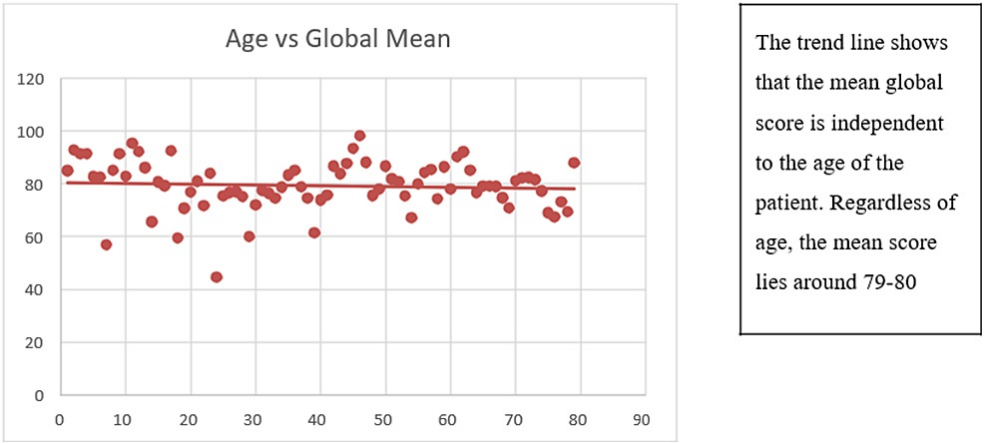
Scatter diagram, correlation of quality-of-life mean score with age

A scatter diagram is drawn to show the correlation of global mean scores with the age of the patient in Figure 2.

Table 3 depicts QoL scores in the patients with respect to the time since they got the transplant. There was a statistically significant difference between the year since the transplant and physical and psychological QoL scores within the groups. As time post-transplant progresses, physical and psychological scores ameliorate. The scores in each domain are higher for patients that underwent transplants three years ago as compared to later. The overall global score is also the highest for patients three years post-transplant. This positive trend may be attributed to fear of complications, aggressive immunosuppression, the burden of increased travel and medication costs during the first-year post-transplant, etc.

**Table 3:** Quality of life scores with time since transplant P-value tests the significance level between the subgroups (e.g., year since transplant 1, 2 and 3 years)

Domains	Year(s) Since Transplant	Mean	Standard Deviation	P-value
Physical Domain	1 year	79.05	10.46	0.035*
	2 years	80.59	11.96	
	3 years	91.43	6.39	
Psychological Domain	1 year	80.37	11.68	0.035*
	2 years	75.29	12.65	
	3 years	88.00	6.50	
Social Domain	1 year	83.85	11.30	0.209
	2 years	80.345	15.34	
	3 years	86.00	20.87	
Environmental Domain	1 year	75.00	12.29	0.310
	2 years	73.19	11.89	
	3 years	81.50	8.02	
Global Mean	1 year	79.57	8.26	0.080
	2 years	77.35	10.87	
	3 years	86.73	4.90	

Tables 4, 5 depict the QoL with respect to gender and marital status respectively. No statistically significant difference in QOL scores was observed in married and single people or with gender. The Global QOL scores are higher in males than in females. Scores are higher in single patients as compared to married patients with the widest gap seen in the physical domain. 

**Table 4:** Quality of life with different genders P-value tests the significance level between the subgroups (e.g., gender-male and female)

Domains	Gender	Mean	Standard Deviation	P-value
Physical Domains	Male	81.05	11.24	0.177
	Female	76.36	9.99	
Psychological Domains	Male	79.51	12.21	0.352
	Female	75.76	11.93	
Social Domains	Male	83.09	13.31	0.574
	Female	80.30	15.09	
Environmental Domains	Male	74.41	11.91	0.567
	Female	76.82	12.70	
Global Score	Male	79.52	9.49	0.445
	Female	77.31	8.47	

**Table 5:** Quality of life scores with marital status P-value tests the significance level between the subgroups (e.g., marital status-married and single)

Domains	Marital Status	Mean	Standard Deviation	P-value
Physical Domains	Married	79.08	10.97	0.137
	Single	83.38	11.35	
Psychological Domains	Married	79.29	11.90	0.594
	Single	77.58	12.90	
Social Domains	Married	83.04	13.52	0.762
	Single	81.97	14.05	
Environmental Domains	Married	74.11	12.19	0.593
	Single	75.68	11.40	
Global Score	Married	78.88	8.95	0.762
	Single	79.65	10.48	

**Figure 3:**
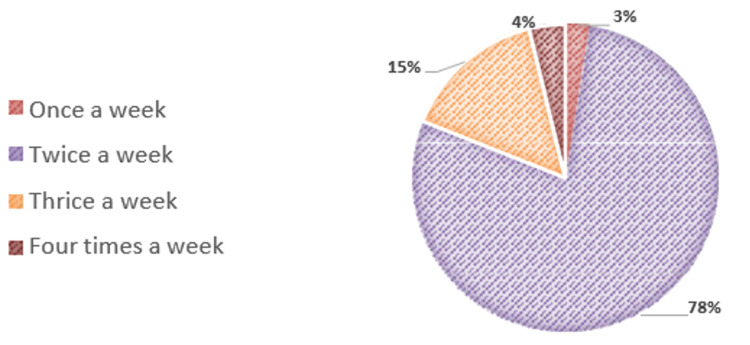
Frequency of dialysis pie chart

As seen in Figure 3, the majority of patients (78%) had undergone dialysis twice a week before transplantation.

No statistically significant difference was noticed between the location of the patient and QoL scores in Table 6.

**Table 6:** Quality of life scores with home province P-value tests the significance level between the subgroups (e.g., Province - Punjab and others)

Domains	Home Province	Mean	Standard Deviation	P-value
Physical Domains	Punjab	80.61	10.32	0.670
	Others	79.51	11.99	
Psychological Domains	Punjab	80.40	12.61	0.232
	Others	77.05	11.74	
Social Domains	Punjab	82.78	14.91	0.851
	Others	83.33	10.82	
Environmental Domains	Punjab	74.94	12.30	0.832
	Others	74.36	11.67	
Global Score	Punjab	79.68	9.94	0.604
	Others	78.56	8.93	

Tables 7, 8 depict that there is no statistical relevance between the QoL scores and socioeconomic status or educational status, respectively.

**Table 7:** Quality of life scores with socio-economic status SE = Socio-Economic P-value tests the significance level between the subgroups (e.g., SE - middle and lower)

Domains	Socio-Economic Class	Mean	Standard Deviation	P-value
Physical Domains	Middle SE Class	81.61	12.61	0.497
	Lower SE Class	79.73	10.46	
Psychological Domains	Middle SE Class	78.02	14.24	0.565
	Lower SE Class	79.76	10.70	
Social Domains	Middle SE Class	81.98	17.31	0.784
	Lower SE Class	82.93	10.57	
Environmental Domains	Middle SE Class	76.80	11.93	0.225
	Lower SE Class	73.33	12.21	
Global Score	Middle SE Class	79.60	11.51	0.781
	Lower SE Class	78.94	7.84	

**Table 8:** Quality of Life Scores With Educational Status P-value tests significance level between the subgroups (eg. education status-matric or below and intermediate or above)

Domains	Education Status	Mean	Standard Deviation	P-value
Physical Domains	Matric or below	79.62	12.81	0.560
	Intermediate and above	81.11	9.43	
Psychological Domains	Matric or below	79.04	12.45	0.973
	Intermediate and above	78.94	12.05	
Social Domains	Matric or below	80.52	16.41	0.179
	Intermediate and above	84.71	9.89	
Environmental Domains	Matric or below	75.79	12.40	0.461
	Intermediate and above	73.78	11.62	
Global Score	Matric or below	78.74	10.48	0.676
	Intermediate and above	79.64	8.24	

**Figure 4:**
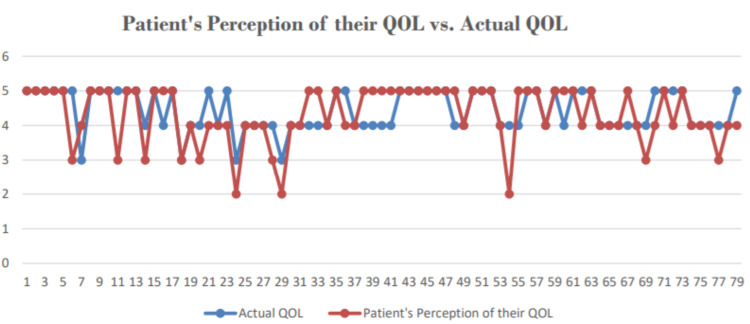
Line diagram representing patient’s perception of quality of life vs. actual quality of life

Figure 4 depicts the differences in perception of QoL and actual QoL. The patient’s perception was taken from the first question of the tool, “How do you rate your QoL”. The two variables are plotted against a Y-axis ranging from 1 to 5, 1 being very poor QOL and 5 being very good QOL. There are only a few noticeable discrepancies. The majority of patients had similar perceptions, with a few exceptions that answered the question more positively as compared to the outcome.

## Discussion

This study analyzed the factors associated with health-related QoL in a representative sample of 79 Kidney Transplant Recipients (KTR) from a urology transplant center in Rawalpindi, Pakistan.

Age

Though not statistically significant, global mean scores in accordance with age showed participants under 35 years of age to have a higher QoL index than those over 35 years. This was in accordance with Von Der Lippe et al. [17], which depicted that patients over 65 years of age actually perceived poorer health-related QoL (HRQOL) in the domain “quality of social interaction” in the conversion from dialysis to renal transplant, while younger patients perceived improvement. One possible explanation for this finding could be that older patients may become more socially isolated after renal transplants than younger patients. Older patients may have fewer personal contacts, and a smaller network of family and friends, and thus participate more infrequently in social activities than younger [18,19].

An assessment study conducted in Bangkok also reported that age was found to be negatively related to the QoL in patients after renal transplants [20]. This may be because as individuals become older, they undergo numerous physical, psychological, and social changes. In particular, physical deterioration leads to a reduced ability to carry out various activities, making them more dependent on others. Thus, they may feel that they are a burden to their family or society. Rajkumar et al. stated that the physical, psychosocial, and sexual impacts of chronic kidney disease on younger individuals are likely to be greater than that for older patients who have less to lose and lower expectations in many aspects of their lives. Consequently, the health improvements and freedoms that transplantation brings to younger patients may have a greater impact than that for more elderly transplant recipients. The participants of the study that were relatively young recipients reported better health a year after transplantation than those who were older in all domains except mental health and emotional wellbeing, in which they showed equivalence.

Time since transplant

QoL scores was assessed with time since transplant and evaluated with the highest global score in patients who had undergone transplant three years back. There was a dip in the mean QoL scores in patients who had the transplant two years back and these participants displayed a lower QoL index than those who had undergone a renal transplant a year back or less. Gross et al. [21] reported that kidney transplant recipients had sustained improvements in mental health but a decline in physical health components one to three years after transplantation. Our study was in concordance with this in that our study, the mental health domain showed improvement, but our study also depicted that the physical domain yielded a significant improvement index which was contrary to Gross et al.'s findings [21]. Nevertheless, both physical and psychological domains had a positive correlation with time since transplant with a p-value of 0.035*, which was significant.

Gender

The slight difference in value could be attributed to the greater stealth and body strength of the male gender as compared to the female gender as well as the difference in lifestyles between the two with one being the labor class and the other having a somewhat sedentary lifestyle in comparison as a major female population of Pakistan resides at home as housewives and/or are unemployed while the male population is usually the sole bread earner but overall it can be seen that gender did not play a significant role in the QoL scores. A huge disparity can be noticed between the number of men versus women receiving transplants. Women were less likely to seek care when it came to their own health but also >30% of the donors were female. As ESRD is equally prevalent in both genders, this inequality could be attributable to inadequate access to health care for females as opposed to males.

The psychological domain showed a slight difference between the two values which can be attributed to the different factors both genders are exposed to. The primary cause for any sort of psychological strain noted was the lack of availability of funds for the continuation of lifelong treatment in the form of medication after the renal transplant. At the same time, the patients did acknowledge the extreme importance of the treatment, and some had even ended up in detrimental property debts just for the sake of preventing the rejection of the transplant. Keeping all these factors in mind, the mean score paints a positive picture in regards to the fact that even while going through the mentioned mental burden the patients maintained a sense of satisfaction and gratitude for being given a second chance at a life that had seemed much more difficult and painful just a few years back.

The social domain included information regarding the personal ties of the patient including their family life as well as relations with friends and acquaintances. The response was strongly positive, and the patients were very satisfied with their different sorts of relationships after the transplant, expressing a sense of heightened affection for them believing they shouldn’t take this extended period of life for granted. Some female patients expressed concerns regarding the duration they had to wait before they would be able to conceive again. 

The environmental domain mainly included information regarding the participants’ ability to enjoy activities of leisure and whether they faced any sort of problems e.g. pain, discomfort, etc. during them. It also included questions about the access and the ease with which they could attain any information regarding their condition as well as their satisfaction with their means of transportation. The lack of a difference between these mean scores between the two genders is somewhat understandable as the effect that environmental factors have on a human being does not involve gender discrimination. Nevertheless, the score also shows a positive outlook of the patients on their healthcare systems and while some patients expressed the difficulty they faced while having to travel on public transports such as buses, vans, and rental cars, their satisfaction with their respective healthcare professions superseded any discomfort they faced. The overall global mean depicted a minute difference in the QoL between both genders following kidney transplantation.

As a whole, it can be concluded that gender did not play a very significant role in the QoL of a patient post-renal transplant. These findings were also in concordance with a study conducted in Bangkok [20] in 2015 as well as Cornella et al. and Liu et al. which reported that gender was not related to the QoL of patients undergoing renal transplants. [22]

Our results were inconsistent with a comparative study conducted in Iraq which portrayed men to have a higher QoL score post-renal transplant as compared to women [23]. In a study by Sayin in turkey, this difference was significant but the rate of life quality in men had been reported considerably lower than in women [24].

Marital status

The sample size again was too small to provide any conclusive results and the main difference in life between these two classes noted was the increased level of sense of responsibility in the married patients, especially those having children. There was not a lot of difference in their QoL from those who were unmarried thus no clear results could be inferred on this. Thus, these results were similar to the study done in Bangkok [20] which reported no significant relationship between marital status and QoL scores. One plausible explanation is that in Pakistani society, individuals tend to live with their extended family, so they have strong family support during their healing process. As a result, they have similar QoL scores regardless of their marital status.

Home location

The overall global mean QoL scores was calculated to be 79.21 while those for the participants residing in Punjab and those residing elsewhere were 79.68 ± 9.94 and 78.56 ± 8.93 respectively. Residents of Punjab were certainly at an advantage within the physical domain considering the relatively small distance that they had to travel to reach the transplant center, where all the participants were scheduled to have their monthly check-ups. This difference is depicted in the results as well, but the value is too small to draw a certain conclusion.

Education

Participants with an education level of intermediate and above had a slightly higher score than those with matric and below education and the difference however insignificant can be mounted up to the different rates of literacy. This was also in accordance with the Thailand study which stated that there was a positive relationship between educational background and QoL scores of patients after renal transplant. This may be because education plays a major role in individuals’ learning, development, and utilization of various resources to equip themselves with self-care efficacy [16-17]. Consequently, in our study, education had a positive effect on patients’ promotion of a good QoL after renal transplant. Patients with higher education reported a better understanding of long-term care, had better access to healthcare and higher incomes and were therefore more likely to display a higher QoL.

**Figure 5:**
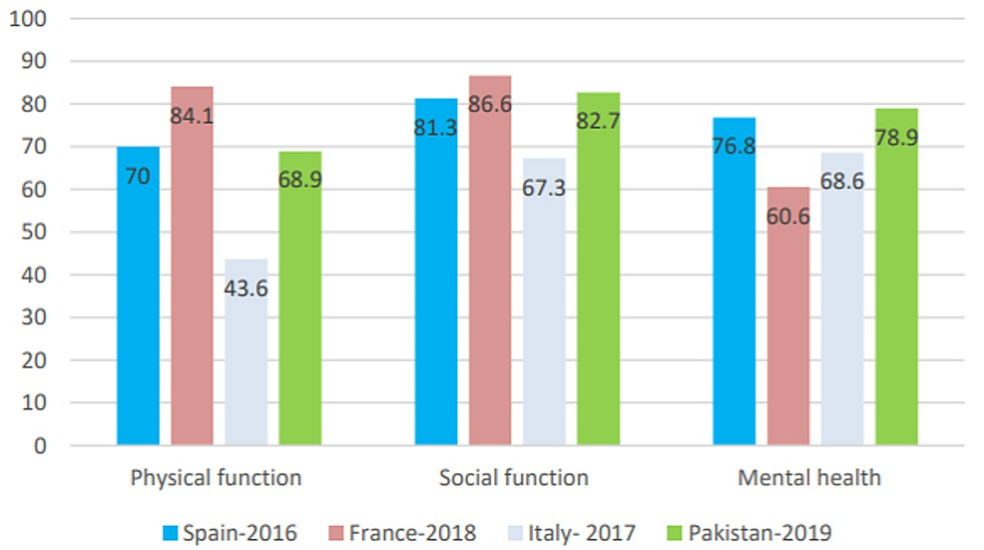
Quality of life scores of different countries vs. of our study

We compared the physical, social and psychological domains of our study with three similar studies conducted in Spain, France [9], and Italy [25]. France had a higher mean global score in the physical and social domains, but it had the lowest score out of all in the psychological domain as depicted in Figure 5. Our study yielded the highest global score in the social domain which can be explained by the presence of cultural, religious, and family support within the southern Asia region as compared to the rest of the world.

## Conclusions

The inferences drawn in this preliminary study will have general relevance to the larger group of patients being treated both in urban and rural setups. Our small and non-random sample cannot yield strong conclusions about the effects of various factors on a patient’s QoL, nevertheless, it does help us track and analyze the overall conditions of these post-transplant patients. In a developing country such as Pakistan, with most of the patients belonging to lower or middle socioeconomic groups, we believe that the patient’s own sense of QoL is overshadowed by the mere exuberance of being given a second chance at life which was portrayed by the discrepancies in the perceived vs actual QoL graph. One common recurring theme that was noticed whilst interviewing the patients was that the difficulties they might have faced post-transplant paled in comparison to how grateful they were to live another day. A positive trend was noticed between the time since transplant and the QoL score which could be attributed to various factors such as the use of aggressive immunosuppressants, fear of injury, fear of transplant rejection, etc. in the first-year post-transplant. Demographic variables such as income, age, location, etc. did not affect the scores of these patients on a great scale. The present study aims to guide clinicians in the improvement of long-term outcomes of renal transplantation in Pakistan and the areas where to focus on the most.

The limitations of our study were that it is a single-center study with relatively low patient numbers, which may have influenced the conclusions and limited the broader application of these results. The majority of the patients were male and belonged to a low socioeconomic group. A better understanding of the QoL of post-renal transplant patients can be made by comparing the pre- and post-transplant QoL scores of a higher patient population whilst also assessing how different comorbid conditions may affect their QoL. Our study lacks the ability to make relevant conclusions when it comes to the QoL of female patients due to less data on gender.
